# A biomechanical comparison between cortical bone trajectory fixation and pedicle screw fixation

**DOI:** 10.1186/s13018-015-0270-0

**Published:** 2015-08-16

**Authors:** Hiroki Oshino, Toshihiko Sakakibara, Tadashi Inaba, Takamasa Yoshikawa, Takaya Kato, Yuichi Kasai

**Affiliations:** Department of Mechanical Engineering, Mie University, Tsu City, Mie Japan; Department of Spinal Surgery and Medical Engineering, Mie University Graduate School of Medicine, 2-174 Edobashi, Tsu City, Mie 514-8507 Japan; Community-University Research Cooperation Center, Mie University, Tsu City, Mie Japan

**Keywords:** Lumbar spine, Biomechanics, Spinal fusion, Spinal instrumentation, Stability

## Abstract

**Purpose:**

There have been several reports on the pullout strength of cortical bone trajectory (CBT) screws, but only one study has reviewed the stability of functional spine units using the CBT method. The purpose of this study was to compare vertebral stability after CBT fixation with that after pedicle screw (PS) fixation.

**Methods:**

In this study, 20 lumbar spine (L5–6) specimens were assigned to two groups: the CBT model group that underwent CBT screw fixation (*n* = 10) and the PS model group that underwent pedicle screw fixation (*n* = 10). Using a six-axis material testing machine, bend and rotation tests were conducted on each model. The angular displacement from the time of no load to the time of maximum torque was defined as range of motion (ROM), and then, the mean ROM in the bend and rotation tests and the mean rate of relative change of ROM in both the bend and rotation tests were compared between the CBT and PS groups.

**Results:**

There were no significant differences between the CBT and PS groups with regard to the mean ROMs and the mean rate of relative change of ROMs in both the bend and rotation tests.

**Conclusion:**

Intervertebral stability after CBT fixation was similar to that after PS fixation.

## Introduction

Spinal fusion using cortical bone trajectory (CBT) was devised by Santoni et al. [1] in 2009. The CBT method of spinal fusion has many advantages compared with pedicle screw (PS) fixation, including a low risk of nerve damage; low invasiveness, as the external side of the intervertebral joint need not be exposed [2]; and high stability among patients with osteoporosis or obesity having spinal diseases [3, 4].

Although several studies have reported that the pullout strength and insertion torque of CBT screws are higher than those of PSs [5, 6], only one study has evaluated the functional spine unit (FSU) and compared intervertebral stability between the two methods (Perez-Orribo et al. [7]), and this study only evaluated intervertebral stability after the CBT method in cases with no damage to the posterior vertebral elements and did not examine the stability of damaged vertebrae. In the present study, deer cadaver models with a damaged lumbar spine were used, and intervertebral stability with use of CBT or the conventional PS fixation methods was evaluated.

## Materials and methods

Twenty lumbar (L5–6) FSUs from a 3-year-old male deer cadaver (Sika deer, *Cervus nippon*) hunted for wild animal damage prevention were used as specimens for this study. The frozen lumbar spine of the deer cadaver was thawed, the muscles and fat were removed, and the upper and lower ends of the specimen were fixed with dental resin. Intact, injured, and fixed models (CBT and PS models) were then created. In the intact model, all the stable FSU elements were preserved.

**Fig. 1:**
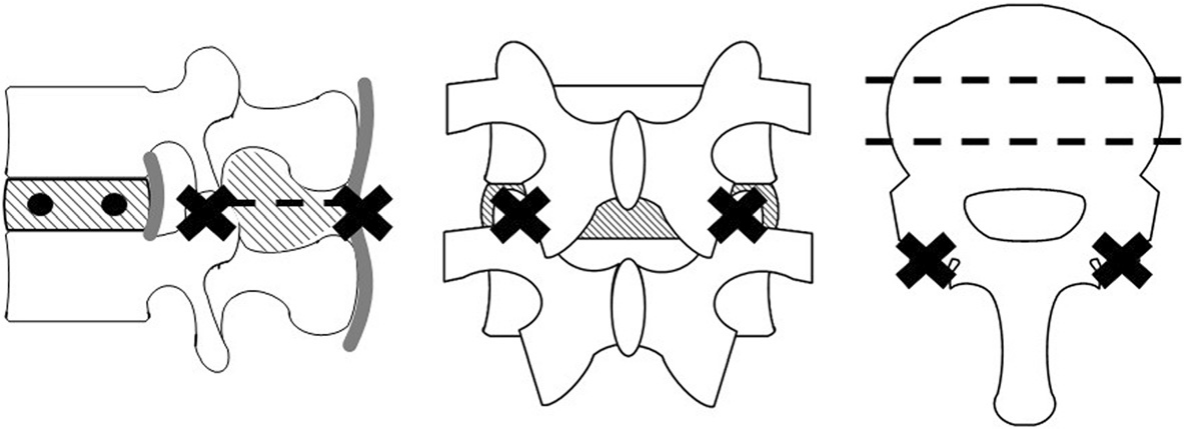
Injured model

Twenty lumbar spines were assigned to two groups: the CBT group that underwent CBT screw fixation (*n* = 10) and the PS group that underwent the usual pedicle screw fixation (*n* = 10). To create the injured model, two holes (3 mm diameter) were drilled into the intervertebral disk (Fig. 1) and the intervertebral joints at L5–6, and the supra- and inter-spinous and yellow ligaments were removed.

**Fig. 2:**
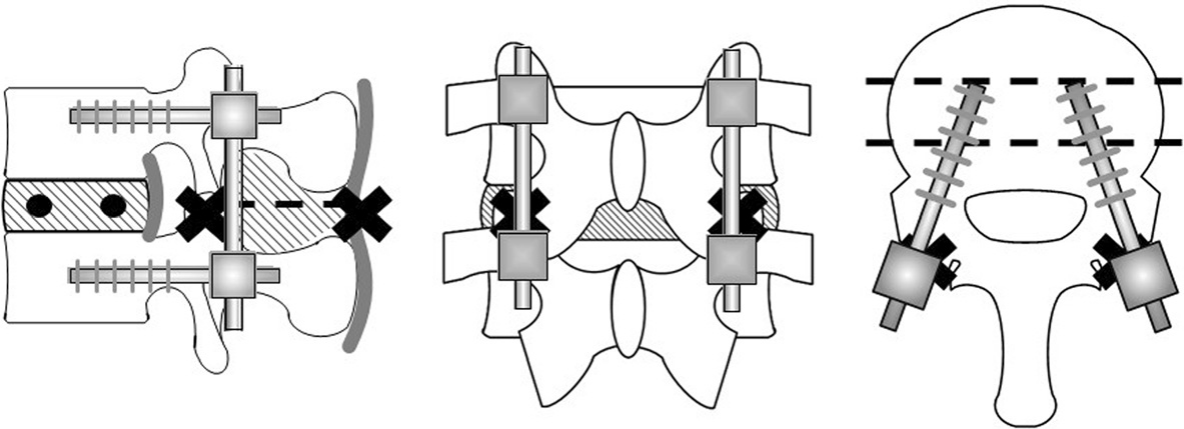
Cortical bone trajectory model

**Fig. 3:**
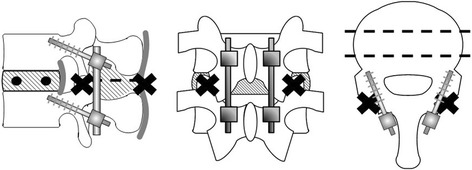
Pedicle screw fixation model

To insert screws into the two fixed models, the authors prepared a burr hole with a 2-mm drill. The burr hole for the pedicle screw was created parasagittally (0°) to the intervertebral disk and at about 15° to the pedicle of the vertebral arch from the outer side to the inner side on the horizontal plane (Fig. 2). The burr hole for the CBT screw was created cranially at about 15° to the intervertebral disk on the sagittal plane and at about 15° to the pedicle of the vertebral arch from the inner side to the outer side on the horizontal plane (Fig. 3). Subsequently, each PS or CBT screw was inserted along the burr holes.

In the CBT group, a screw 4.5 mm in diameter and 25 mm in length (Medtronic Sofamor Danek: Solera 4.75, Memphis, TN, USA) was used, and in the PS model, a screw 6.5 mm in diameter and 30 mm in length (KiSCO: S-Line II, Saint-Priest, France) was used. With respect to the depth of insertion of the screws, since the CBT and pedicle screws could rupture the wall of the vertebral body of the deer spine at depths of 20 and 25 mm, respectively, the depth of insertion was set at 15 and 20 mm for the CBT and pedicle screws, respectively. Although the screw may project from the spine, the size of the projection was 10 mm for both the CBT and pedicle screws, and the lever arm was the same in both groups; thus, it was possible to compare both biomechanically.

**Fig. 4:**
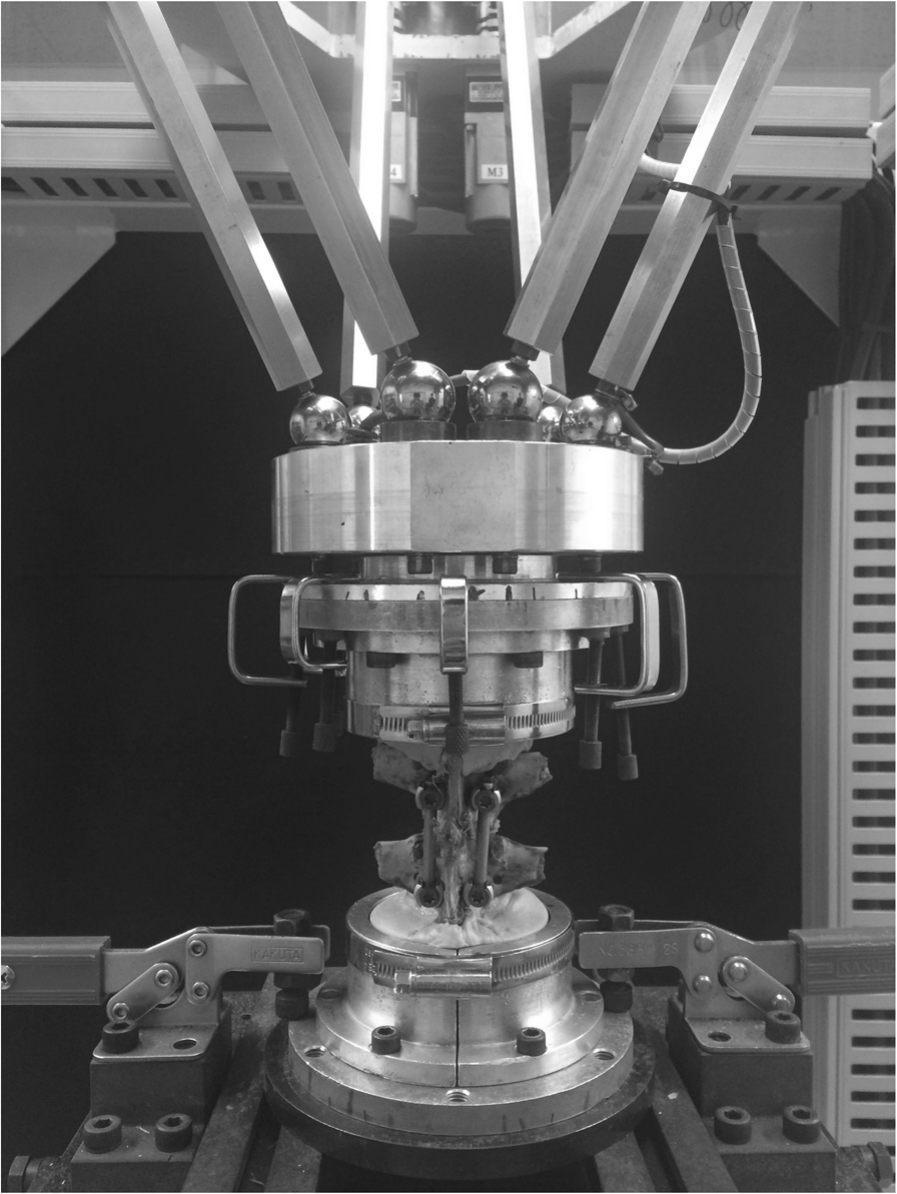
Six-axis material test machine developed in our laboratory

For the test, a six-axis material testing machine [8–10] developed in our laboratory (Fig. 4) was used. This testing machine adopts a parallel mechanism. A set of two actuators are located parallel at 120° to the object, and each of the six actuators is independently controlled. At the hand side, a six-axis kinesthetic sensor is equipped to detect the force in the *x*-, *y*-, and *z*-axes and the torque around each axis. Furthermore, this kinesthetic sensor enables force control by feeding back the detected values to the control system and enables motion with any degree of freedom.

Using this testing machine, bend and rotation tests were conducted on each model. In the bend test, eight directions were measured: anterior, antero-right, right, postero-right, posterior, postero-left, left, and antero-left. In the rotation test, two directions were measured: right and left rotation. The torque was set at 3.0 Nm for the bend test and 4.0 Nm for the rotation test. In the bend test, the number of degrees of freedom was set to 3 to allow genuine bending in one plane. In the rotation test, the number of degrees of freedom was set to 4 to allow displacement along the *x*-, *y*-, and *z*-axes and rotation around the *z*-axis. Since displacement owing to coupling motion should be prevented to determine pure bending or rotational angle in one place, 3 or 4 of the 6 degrees of freedom were prevented by the six-axes material testing machine.

Angular displacement from the time of no load to the time of maximum torque was defined as range of motion (ROM). The rates of relative change of ROMs between the injured model and each fixed model (CBT or PS) were calculated using the following formula: {(ROM in the fixed model − ROM in the injured model)/ROM in the injured model} × 100. The mean ROMs of the intact, injured, or fixed model for both the bend and rotation tests and the mean rates of relative change of ROMs for both the bend and rotation tests were compared between the CBT and PS groups using a Mann-Whitney test. *P* < 0.05 was considered significant. This study was performed with the approval (No. 1543) of the Research Ethic Committee in Mie University Graduate School of Medicine.

## Results

### Bend test

**Fig. 5:**
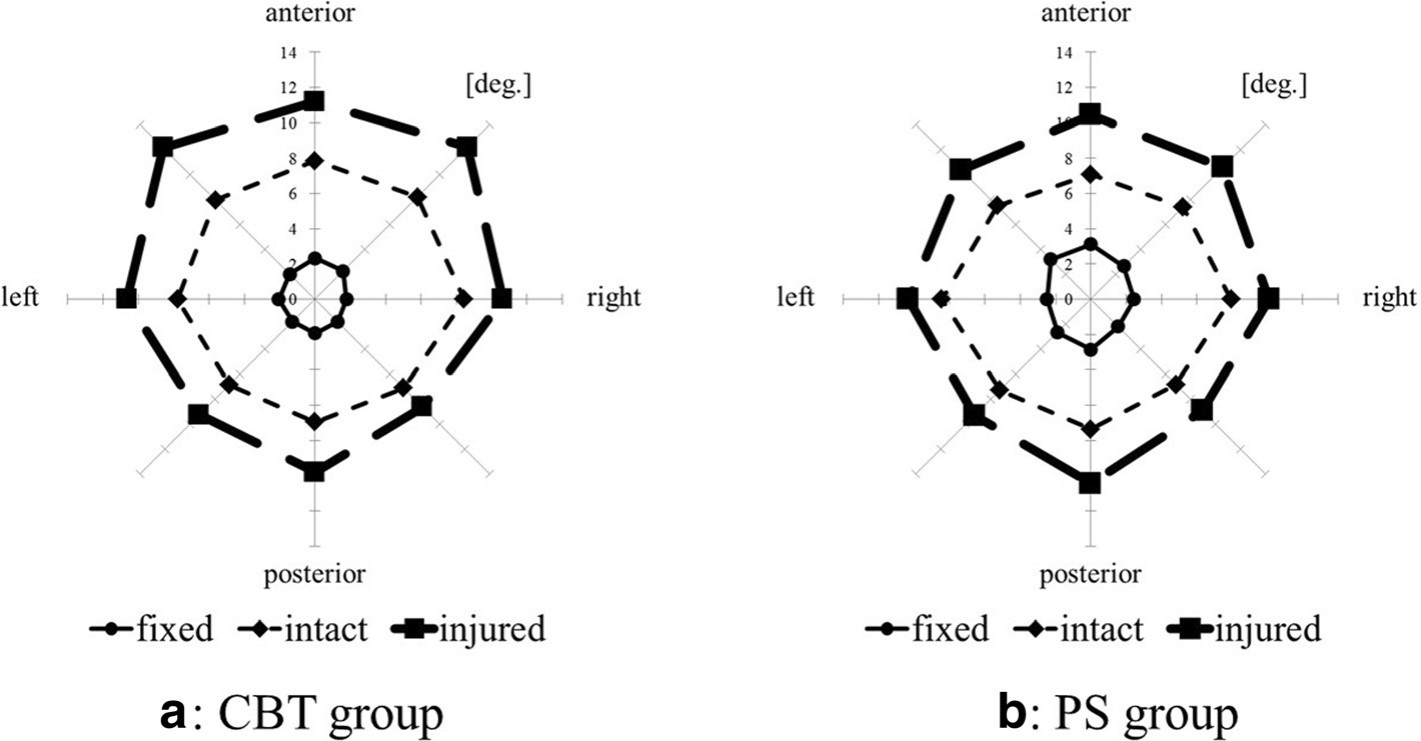
Mean ROMs of bend test. **a** CBT group. **b** PS group

The mean ROMs of the intact and injured models of both the CBT and PS groups in each of the eight directions in the bend test are shown in Table 1 and Fig. 5. At the results of mean ROMs in the intact and injured model, there were no significant differences between the two groups. The mean ROMs of the fixed model of both the CBT and PS groups in each of the eight directions in the bend test are shown in Table 1 and Fig. 5. The mean ROMs in the CBT group ranged from 1.8° to 2.3°, and the mean ROMs in the PS group ranged from 2.2° to 3.2°, indicating that mean ROMs in the CBT group were slightly lesser than those in the PS group. However, there were no significant differences in the mean ROMs between the CBT and PS groups. In all eight directions, the mean rates of relative change of ROMs were slightly lower in the CBT group than in the PS group (Table 2); however, there were no significant differences in the relative change between the two groups.

**Table 1:** Mean ROMs of the bend and rotation tests

		CBT (°)			PS (°)		
		Intact	Injured	Fixed	Intact	Injured	Fixed
Bend test	Anterior	7.8 ± 1.8	11.2 ± 2.4	2.3 ± 0.9	7.1 ± 2.1	10.4 ± 3.3	3.1 ± 1.8
	Antero-right	8.2 ± 2.0	12.2 ± 3.2	2.3 ± 0.9	7.4 ± 1.8	10.6 ± 2.7	2.6 ± 1.7
	Right	8.4 ± 2.2	10.6 ± 2.7	1.8 ± 0.5	7.9 ± 2.1	10.1 ± 1.2	2.4 ± 1.4
	Postero-right	7.1 ± 1.1	8.6 ± 1.6	1.8 ± 0.5	6.8 ± 1.3	8.9 ± 2.1	2.2 ± 0.8
	Posterior	7.0 ± 1.2	9.8 ± 1.9	1.9 ± 0.8	7.4 ± 2.1	10.4 ± 3.5	2.9 ± 1.4
	Postero-left	6.9 ± 1.5	9.3 ± 2.4	1.8 ± 0.5	7.3 ± 1.7	9.3 ± 1.4	2.7 ± 1.2
	Left	7.8 ± 2.1	10.6 ± 3.1	2.1 ± 0.7	8.4 ± 1.9	10.3 ± 2.1	2.5 ± 1.7
	Antero-left	8.0 ± 1.7	12.2 ± 3.2	2.0 ± 0.5	7.5 ± 1.6	10.4 ± 2.0	3.2 ± 2.1
Rotation test	Left	1.4 ± 0.6	7.0 ± 2.0	2.7 ± 0.9	1.5 ± 1.0	7.3 ± 2.1	3.0 ± 1.7
	Right	1.4 ± 0.7	7.0 ± 2.0	2.7 ± 1.0	1.5 ± 0.8	7.1 ± 2.0	3.2 ± 1.7

There were no significant differences between the CBT and PS groups

**Table 2:** Mean rates of relative change of ROMs in the bend or rotation test

	CBT model (%)	PS model (%)
Bend test		
Anterior	−79 ± 8	−70 ± 15
Antero-right	−81 ± 5	−74 ± 15
Right	−83 ± 4	−76 ± 12
Postero-right	−79 ± 3	−76 ± 6
Posterior	−81 ± 5	−73 ± 12
Postero-left	−80 ± 5	−72 ± 10
Left	−80 ± 6	−76 ± 15
Antero-left	−83 ± 5	−68 ± 21
Rotation test		
Left	−62 ± 11	−55 ± 18
Right	−61 ± 11	−51 ± 16

There were no significant differences between the CBT and PS groups

### Rotation test

The mean ROMs of the intact, injured, or fixed models of both CBT and PS groups in each of the two directions in the rotation test are shown in Table 1. The mean ROMs in the CBT group were slightly lesser than those in the PS group; however, there were no significant differences in the mean ROMs between the groups. The mean rates of relative change of ROMs in both rotation directions were slightly lower in the CBT group than those in the PS group (Table 2); however, there were no significant differences in relative change between the two groups.

## Discussion

The CBT method is useful for lumbar spine fusion in patients with osteoporosis or obesity, and studies by Song et al. [3], Ueno et al. [4], Takata et al. [11], and Mizuno et al. [2] have shown excellent results without any complications using the CBT method, although the evaluation period of these studies was very short. Rodriguez et al. [12] stated that, using the CBT method, the screw can be inserted into vertebral bodies in which the PSs are already inserted; thus, the CBT method is useful in repeat surgery of the vertebrae.

The primary biomechanical evaluations of the CBT method included the pullout test of the inserted screw and the insert torque. Santoni et al. [1] reported that the pullout strength increased by 30 % compared to that with the conventional PS, and Matsukawa et al. [5, 6] reported that the insertion torque increased 1.7-fold. The toggle test results indicate that the use of the CBT method results in a significantly higher stability than the PS fixation method [13]. Furthermore, screw insertion using the CBT method has been reported not only for the lumbar spine but also for the thoracic spine. Perez-Orribo et al. [7] examined fixation of the FSU in a human cadaver lumbar spine and found that the CBT model had equivalent stability to the PS model in the bend and rotation tests, regardless of the presence of interbody fusion. The current study is the first to compare intervertebral stability between the CBT method and that with the PS method for damaged both anterior and posterior vertebral elements.

There has been a concern that the CBT method results in low intervertebral fixation biomechanically because the screws used in this method are shorter than the PSs, but since there were no significant differences in the stability between the groups in the present study, we suppose that the CBT screw may have a larger contact area with the cortical bone, thus increasing the efficacy of the screw. Therefore, the CBT method may result in good fixation in patients with decreased bone quality, such as those with osteoporosis. During the operation, moreover, since the insertion point of the CBT screw is inside that of the conventional PS method, it is considered that deployment of the posterior muscle group can be minimized, and the amount of bleeding can be reduced. Since the insertion point of CBT screw is more caudal than that of the PS screw, avoiding iatrogenic facet and articular capsule injury and reduction of adjacent intervertebral injury can be expected.

The limitations of this study include the fact that deer spines were used as specimens, the insertion angle of the screw in the sagittal/horizontal plane was not always constant, and the present injury model was not created assuming decompression in humans.

Wasinpongwanich et al. [14] reported that although intervertebral disc height, vertebral body size, and intervertebral joint shape differ between deer and human spines, deer lumbar spines can be used without any problems in experiments to measure instability after the FSU is destroyed or to check stability after fusion with an implant. However, use of human vertebrae is preferable; therefore, we will perform a similar experiment using human cadavers in the future.

The positions of the screws after insertion were not confirmed using radiography, but in the present study, since the screws were inserted while monitoring each sample from a 360° radius, it is likely that the screws were generally inserted in the appropriate direction.

In the present injury model, when partial resection of the facet joint was performed, the degree of instability may have differed largely among the samples, but if the facet joint was totally resected, the same intervertebral instability could be expected for all samples. If the spines of human cadavers are used, it is important to prepare the model appropriate to clinical practice, but since deer cadavers were used in this study, the instability of each sample was standardized by completely resecting the facet joint and preparing a highly unstable model.

## Conclusion

Deer cadaver models with a damaged lumbar spine were used to evaluate intervertebral stability after CBT fixation or conventional PS fixation methods. There were no significant differences in mean ROMs and mean rates of relative change of ROMs in both the bend and rotation tests between the CBT and PS groups. Therefore, intervertebral stability after fixation by the CBT method was similar to that after fixation by conventional PS fixation.
