# Erratum to: A biomechanical comparison between cortical bone trajectory fixation and pedicle screw fixation

**DOI:** 10.1186/s13018-015-0295-4

**Published:** 2015-09-24

**Authors:** Hiroki Oshino, Toshihiko Sakakibara, Tadashi Inaba, Takamasa Yoshikawa, Takaya Kato, Yuichi Kasai

**Affiliations:** Department of Mechanical Engineering, Mie University, Tsu City, Mie Japan; Department of Spinal Surgery and Medical Engineering, Mie University Graduate School of Medicine, 2-174 Edobashi, Tsu City, 514-8507 Mie Japan; Community-University Research Cooperation Center, Mie University, Tsu City, Mie Japan

## Erratum

After publication of the original article [1] it came to our attention that the legends for figures 2 and 3 had been interchanged. The correct legend for figure two is ‘Pedicle screw fusion model’ and the correct legend for figure three is ‘Cortical bone trajectory model’.
